# Selections and Abstracts

**Published:** 1900-12

**Authors:** 


### *SELECTIONS AND ABSTRACTS.*

---

### An Adamless Eden.

The commencement of the twentieth century witnesses the most startling discovery ever made in the scientific world, *i. e.*, the *creation of life* by chemical means. It was a Chicago discovery, too, and Professor Jacques Loeb, of the University of that city, has added more laurels to those he has already obtained. Dr. Loeb has demonstrated, if we are to believe the statements now admitted in laboratory circles, that artificial parthenogenesis is possible among mammalians, and certain changes in the ions of the blood may secure animated beings from the higher natural orders, including the highest type of all, *man himself*, or rather let us say *woman*, for if the Loeb theory becomes practical no men will be needed to run the affairs of the earth. Women, once initiated in laboratory work, can proceed to populate the land without any carnal intercourse with man. Any virgin or spinster can start her laboratory kindergarten on an exclusive female basis; any male developed from an ovule can be promptly destroyed, so that the entire crop derived by artificial parthenogenesis may become femininized. It is even claimed by such theological experts as the Reverend R. S. McArthur that if Loeb's theory be proved "it would merely illustrate the method by which God secured the glorious result." It will be noted from this that the Reverend Doctor McArthur admits that any priority in discovery belongs to a higher power than Professor Loeb of the Chicago University. It is well to remark that science and religion always go hand in hand at the Chicago University.

It is true that there is a vast difference between the sea-urchin placed in sea water, to which the solution of chloride of magnesium is added to increase osmotic pressure, and the ovule of the human urchin deprived of the contact of the fertilizing spermatozoid. Yet Professor Frazier McIntosh assures us that "there is no difference in the life that animates the sea-urchin and the human being," and adds: "It is merely a question of less and greater development or evolution. In an earlier period of evolution we were sea-urchins. Before that we lived in a still more primitive guise, and so we go back to the germ that was the beginning of all life. This experiment tends to show that that germ was the result of the chemical action of lifeless substances."

When Charles Kingsley wrote his "Land Babies and Water Babies" he never once dreampt that sea-urchins and land-urchins would be raised and propagated by means of the electrolytic salts added to sea water. We are assured that when Professor Loeb adds cane-sugar that does not contain electrolytes, the eggs are ultimately killed. It will be remembered that a celebrated German scientist, lately expelled from a Vienna university for claiming he could regulate the sex among vertebrates by saccharine substances, clearly pointed out that saccharine matter had certain physiological actions in genesis that controlled sex conditions, and here comes Professor Loeb and claims that while the eggs of the sea-urchin were developed, they were ultimately killed by cane-sugar, thus proving on a scientific basis that electrolytes are not absolutely essential in artificial parthenogenesis.

Yet, putting aside all *persiflage,* Professor Loeb's discovery affords a vast field for contemplation, and there is no end to the possibilities of life creation as viewed through laboratory spectacles. There is no more reason why the protoplasmic cell from which human kind evolved might not as well be brought into ordinary terrestrial surroundings as the spine-backed sea-urchin. This discovery of Professor Loeb's fits in exactly with the neuron doctrine, that will presently start a new school of medical scientists, the neuropaths, who will throw all the germ theory of disease into the shade, and go, with a meteor-like light into the medical heavens, to making the spark of life for all humanity instead of dealing in abstruse calculations of a microbian kind—the mere reflections on death. It is easy to discern this new twentieth century comet of medicine coming; it appears in the zenith over the Chicago University. It is the development of the egg without fertilization, the biological parthenogenesis attached to the medico-doctrinal theory of the neuron. The medical man twenty years hence who does not believe in the new school will be only comparable to the modern physician who scoffs and derides the microbian theory of disease. Life and death! Who would fail to choose between the absolute microbian theory of disease and the Loeb theory of parthenogenesis when attached to the truly scientific doctrine of neurons.

The *American Journal of Physiology* contains the technical report of Professor Loeb. To any one familiar with chemico-biological work the method must appear very simple. We really believe Professor Loeb's sea-urchins were of laboratory origin. Yet many varieties in the lower animal scale, so far as is known, indulge in parthenogenesis without the use of chemicals, at least only of those derived from Nature's laboratory. The more I read on this subject, Mr. Editor, the more I feel like dilating, but there is a limitation as to space in most well-regulated journals like the *Lancet-Clinic.* We only suggested at the start that, if Professor Loeb's work is extended, in these days of the higher feminine culture, womankind being vastly superior to man, now that babies can be raised without the terrors of matrimony and the pains of maternity, this world, by say the twenty-fifth century, will no longer have any men. It will be an Adamless Eden, the children only of feminine protoplasms being raised. Of course, the *savants* of Chicago University will be able in time to devise some new method by which the primitive protoplasm of the female ovule may be obtained in quantities sufficient to propagate the human species of a sole feminine type. We always knew that woman would some day get along without man. The cigar and cigarette fiend, the wine-bibber aud boozer, the masculine gay deceiver with the too available spermatozoids, will be a thing of the past in a few short centuries. The historical masculine names will never be regarded as anything but myths, in the times of the ancient gods. There will be no night-keys nor latch-holes to put them in at the inebriated midnight's lonely hour. Perhaps in some museum of antiquities like that of the great Boulak collection of ancient Egyptian mummy cases and clothing, some pair of old pantaloons may be found around which the sweet maidens of the twenty-fifth century will gather, those fair daughters of the artificial parthenogenesis order, who will read with wondering eyes the inscription—"Jones's breeches, nineteenth century, commonly attributed to what was once called man, a sexless creature destroyed by science." Of course, the love-stories and novels will no longer have a place among the cultured followers of the future. There will be no lawyers, no doctors, only *females*. It is a survival of the fittest, after all.

An old maid patient of mine up Buck Creek, who has been reading the Cincinnati and Chicago dailies, informed me that she had sent to Chicago University for a 10–8n$NaCl_2$ solution, and was going to try it on an unfertilized ovum. I asked her where she was going to get the ovum, and she answered me curtly; however, she has not passed her menopause, and ranked high in her biological studies at Vassar.

But avaunt protoplasm! Come up and take that hunt with me. The Kankakee is not far from Galensville, and you remember the song:

"Mark overhead! A canvasback!
Mark! mark! a flock of teal!
And swiftly on each flying track
Follows the shotgun's peal.
Thus rolls that call till twilight's tide
Rolls in like some gray sea,
And whippoorwills complain beside
The lonely Kankakee."

Be sure and bring a bird dog along, and we will discuss other things than parthenogenesis.—*Timothy Tabbs, M.D., in Cincinnati Lancet-Clinic.*

---

### PHOTO-THERAPY.

The bactericidal action of sunlight has long been known and during the last two years considerable experimentation has been done in the treatment of various parasitic diseases by concentrated light, both the direct rays of the sun and the rays from the electric arc light having been used. Finsen of Copenhagen has done the most and the best work in this line, and a full report of his experience may be found in the *British Medical Journal* for September 30, 1899. The same article was published simultaneously in the *Philadelphia Medical Journal*. For some years Finsen had been treating smallpox beneath red glass, on the theory that by excluding the chemical rays of the sun there would be less irritation of the skin and consequently less danger of pitting. He found by experiments that the chemical rays have a decided power of penetrating the skin as well as a marked bactericidal action, the latter being especially the case with the blue and the violet rays. The best results were obtained by excluding the red, yellow and green rays and using as many as possible of the violet and ultra-violet rays. The direct sunlight must be condensed, as otherwise it is not strong enough. Finsen used a hollow plano-convex lens filled with a blue solution to exclude the red, yellow and green rays. The treatment lasts from half an hour to an hour every day. Some reddening of the skin around the lupus patch (for the treatment has been mostly used in lupus) is noticed, but no cauterizing effect. The treatment is painless. His results were almost uniformly good, although it remains to be seen how permanent they are. Abrams of California and Kime of Iowa have both reported favorably upon this method of treatment. Kime has devised (*Iowa Medical Journal*, April, 1900) a compound reflector thirty inches in diameter, each part set at such an angle that the sunlight falling upon the whole reflector is focused at a point eight feet in front of it upon an area six inches in diameter. He claims good results from its use. The whole subject of photo-therapy is in its infancy, but we look for very valuable results from continued investigation of it. The writer has been so well satisfied with the results of the treatment of lupus with superheated air, that, while he would welcome a better treatment, he would have to be pretty thoroughly convinced of its superiority before adopting it. It has also been claimed for concentrated light that its penetrating power is so great that pulmonary tuberculosis and other diseases caused by micro-organisms in the deeper structures of the body may be cured by its use, and that as all the blood circulating in the body passes several times through the area bathed in the light, any micro-organisms in the blood will be destroyed. While not prepared to deny that such may be the case, we do not consider the evidence at present available as at all convincing. The subject however is one well worth careful study, and we shall await with great interest the results of further research, which will doubtless be forthcoming in the immediate future. It is believed by some scientists that the great motive power of the future lies in the energy of the sun's rays; it is by no means impossible that from the same source may be derived our most valuable therapeutic agent.—*St. Paul Med. Journal.*

---

### ANKLE SPRAINS.

Edward H. Ochsner, in speaking on this subject before the Illinois Medical Society, as reported in the *Journal of the American Medical Association* for June 2d, described and demonstrated a method which consists in careful and systematic strapping with rubber adhesive straps. These are cut from one-half to three-quarters of an inch in width, and the proper length. If a small ankle, they should be one-half inch wide; if a large one, they may be three-quarters of an inch, but no wider; on this and on the accuracy with which they are applied depends the success of the method. If the straps are too wide, or if they are applied in a haphazard manner, failure is sure to result. The foot is held at slightly less than a right angle and a trifle everted; the former element in the position is observed because it is easier to walk on a painful ankle if it is held slightly in the calcaneum than if held in the equinus position; the latter element is observed because ankle sprains are usually caused by a sudden inversion of the foot, thus injuring the external ligaments; hence slightly everting the foot relieves the tension of these ligaments and places them at rest. With the foot in this position, one end of the strap is applied to the inner surface of the foot near its posterior end, and brought under the heel and up on the outer posterior surfaces of the leg to within a few inches of the knee. At the lower end this falls into the depression just posterior to the external malleolus. A shorter strap is now applied by placing one end to the inner surface of the heel near the sole of the foot, then bringing it around over the tendo-Achillis to the outer surface of the foot, making it cover the first strap at a right angle and passing along parallel to the under border of the sole of the foot, then over the dorsum of the little toe. Another long one is then applied, anterior to the first, overlapping it about one-third of its width; then a short one, and so on alternately until the outer anterior aspect of the ankle is reached. Over all this a hard-rolled bandage is carefully and snugly applied. The patient is directed to lie still with the foot elevated until the warmth of the body has caused the plaster to adhere firmly. In a great majority of instances the patient can walk, with reasonable comfort, after a few hours. He reported four cases from among the number he has treated, because they illustrated four different classes. The treatment of ankle sprains by this method he has found eminently satisfactory.—*The Charlotte Medical Journal.*

---

### STANDARDIZATION OF DRUGS.

What right has any firm, whose business is to furnish the physician with his principal weapons, to place upon the market pharmaceutical preparations of unknown medicinal value? Should we not expect, yes, even demand, that the producer of fluid extracts make his products conform to some standard of excellence—that he shall indicate what effects his fluid extracts may be expected to have ere he sends them forth from his laboratory?

It has been shown that even drugs selected with care vary most extraordinarily in their percentage of active principles. Witness, for example, this statement by the editor of a leading pharmaceutical journal,* who knows whereof he speaks:

"Professar Puckner assayed nineteen samples of belladonna leaves, procured, mind you, from dealers who were told that only the best was wanted, and that purchase would depend upon the results of assay. He found these nineteen samples to range in alkaloidal content from .01 to .51 per cent.! The strongest sample fifty-one times as strong as the weakest."

The most careful treatment of such drugs, with the choicest menstrua, and by the most approved processes, will yield preparations that may be fair to look upon, but in medicinal value they will vary just as much as the crude drugs from which they are

*Bulletin of Pharmacy, January, 1899.

made. The compensatory remedy for this unfortunate condition is standardization—chemical standardization when practicable, and when that method is inadmissible, as it often is, physiological standardization. It has been found that certain important drugs cannot be assayed chemically, as their medicinal virtues reside in unstable bodies, and these are readily decomposed in the analytical processes. For this reason the strength of such powerful and useful drugs as digitalis, aconite, convallaria, strophanthus, ergot, cannabis indica and many others can not be determined satisfactorily by the analytical chemist. However, the problem which proved to be an insurmountable difficulty to the chemist was solved by the pharmacologist with ease. He tests upon living animals all drugs that cannot be assayed chemically. Dogs, rabbits, fowls and guinea-pigs, receive doses of the preparations under examination. Accurate observations of their physiologic effects are made, variations are noted and corrected, until the preparations correspond in medicinal strength with the adopted standard extracts.

Formerly the physician was obliged to make his own physiologic tests of ergot, digitalis, and so on; not upon dogs and guinea-pigs, however, but upon his patients. The old way was to begin with small doses of powerful drugs, and then to push them until the desired effect was produced. The new way is a much better one; it is safer for the patient, more satisfactory to the physician, and it is more scientific. Prompt results are assured, for the physician knows just how much fluid extract of ergot, aconite or cannabis indica he need include in his initial dose to secure a definite result.

The name of the greatest pharmaceutical manufacturing house in this country is so closely linked with the phrase, "drug standardization," that the mere mention of one suggests the other. Parke, Davis & Co. began years ago to manufacture a full line of standardized fluid extracts that are guaranteed to be of definite and uniform strength. More recently they devised and perfected methods for standardizing physiologically those important drugs that are incapable of analysis by chemical processes. Parke, Davis & Co. have done a great deal for the medical profession and for humanity, and standardization, more especially physiological standardization, is one of their greatest achievements.

### HUSBAND'S LIABILITY FOR CARE OF INSANE WIFE.

The Supreme Court of California has rendered decisions in two cases brought by the St. Vincent's Institution for the Insane *vs.* John T. Davis. One was for boarding and clothing his insane wife prior to June, 1894, and the other was for keeping and caring for her, providing for her suitable boarding, lodging, clothing, washing, medicine, and medical and other attendance after that date. In both cases, the court cites section 174 of the California Civil Code, which provides that, when a husband fails to make adequate provision for the support of his wife, then—except in certain cases—any person may supply her with necessaries, and recover the value thereof from the husband. Under this rule, applied to the different circumstances of the two cases, in the first-mentioned case the Supreme Court affirms a judgment in favor of the St. Vincent's Institution for the Insane, and, in the other case, in favor of the husband. In the first case, there was a very strong presumption that the husband had left his wife in a small town in Illinois, intending that her identity should be lost, that she might no longer be a charge upon him. Under such circumstances, the court holds the husband would be liable for necessaries, even though the parties supplying them did not know of his existence, or that the woman was married. And with regard to the contention that the evidence was insufficient to sustain a finding that the service was rendered on the credit of the husband, it being argued that it did not appear that he was even aware that she was being kept and provided for by the institution at all, the court answers that, even if he had no such knowledge, it would not follow that he was not liable, or that the service was not rendered at his request. But, in the second case there was a finding that the husband, desiring in good faith to care for his wife elsewhere, had, in June, 1894, demanded of the institution that she be delivered to him, and that the institution, without legal cause or excuse, had refused to comply with such demand, and against the will of the husband had thereafter retained the wife in its custody. Such being this second case, the court holds that no recovery for keeping and caring for her could be had therein, the section of the

Civil Code quoted requiring that whoever supplies necessaries to a wife must, in order to recover therefor, show neglect on the part of the husband to make adequate provision for her support, which was not shown in this case, it not being enough for the purpose that some suspicion was cast upon the motive of the husband, there also being some evidence tending to show good faith on his part.—*Journal A. M. A.*

---

### SUSPENDING TRIAL FOR EXAMINATION BY PHYSICIAN.

One of the grounds on which the Supreme Court of Georgia was besought in Herndon *vs.* State, to reverse a conviction of murder, was that the trial court had refused to allow a medical expert, who had been introduced by the accused, to examine an indenture or depression in the skull of the accused, and then testify as to the effect it would produce on his mind. In his statement to the jury, the accused had stated that the depression had been made in his early youth by a falling stone, that he had subsequently had a severe case of typhoid fever, and that since then he had been at times ignorant or unconscious of what he said and did. But the matter of suspending the trial for the purpose mentioned, the Supreme Court holds, was entirely within the discretion of the trial court. It says that a trial court may or may not suspend a trial for such a purpose, according to the circumstances of each particular case, and that where a matter of practice is within the discretion of the trial court, the Supreme Court will not interfere unless such discretion is manifestly abused. Continuing, it says it can not establish any fixed rules to govern courts in this respect. In some cases an examination of an injury might be made in a few minutes. In others, hours might be consumed before the expert could come to any definite conclusion as to the nature and character of the injuries. Besides, it makes a point here of the fact that this appeared to have been the second trial of this case, and that the accused and his counsel had had abundant opportunity to have had the examination made before the trial. Had he done so, then the expert, it holds, could have testified as to his opinion of the effect the injury would have produced upon the mind of the accused.—*Journal A. M. A.*

### CHOLERA IN THE EAST.

In a special report, the U. S. Consul at Bombay says that cholera is raging in India, that it is of a very fatal type, prevailing among all classes in all sections of the city, and generally throughout the famine area of Western India. Eight hundred and thirty-four cases and 751 deaths were reported for the week ending August 22 in the city of Bombay alone. In addition to the above official reports, the press reports state that there have been 4,500 deaths from this disease in Kabul, Afghanistan. It is therefore likely that it will soon be necessary for health officers to give special attention to vessels coming from ports of the Black Sea, Persian Gulf and the Red Sea. In this connection, the report of the commission of the Marine-Hospital Service, appointed in 1892 to investigate the cholera epidemic and the danger of contagious diseases from foreign countries, is of particular interest at this time. This report, with maps showing the usual routes of cholera, is published in full in the annual report for 1893.

Hurdwar, in India, where a great annual fair is held, seems to be the starting distributing point, for it is to Hurdwar that traders come from all parts of India to meet a like stream from Persia and Afghanistan, where they exchange the products of the two countries. Hurdwar is on the Ganges, and the religion of the Hindoo makes it obligatory to bathe in the stream at this point, and when there is a drouth the stream is nothing but a series of pools, in which every one must bathe. It becomes a focal point from which disease radiates. Then along the routes of travel the disease spreads from India to Afghanistan, thence to Persia, thence into Turkey in Asia, Turkey in Europe, and thence to other portions of Europe and the civilized world.

---

### SIMPLE OPERATION FOR HEMORRHOIDS: ENUCLEATION.

Dr. J. Rawson Pennington, Chicago, contributed this paper, which was profusely illustrated. He gives a cathartic the second night before the operation, a saline the following morning and a bath, and colonic flushing the night before. The next morning he gives an enema of from one-half to one pint of cool water, and operates two hours later. He emphasizes the importance of carefully examining the entire rectum. He grasps each anal quadrant at the muco-cutaneous junction with a pair of forceps; the anus is everted, and the internal tumors exposed. Seizing with the hand the forceps attached to the posterior quadrant, he fully everts it, and, with a pair of scissors curved on the flap, cuts off the redundant membrane only, which is usually about one-third or one-half of the uppermost part of the hemorrhoidal node. This permits the blood in the tumor to escape. All of the angiomatous tissue is carefully removed, when the remaining wall collapses. Each quadrant in regular order is treated in a like manner. A stream of hot sterilized water flows over the field continuously during the operation. Spurting vessels, if any, are caught with forceps and thoroughly twisted. Should this fail to control hemorrhage, he throws a ligature around the vessel and ligates it. The operation having been completed, he introduces a rubber-covered tampon, which has been fully described in previous articles by the author.

The advantages of this method are: There are no stumps to slough, nor nerves caught and squeezed, which will cause excruciating pain, as when the ligature is used; nor are the nerves and tissues burned to a crisp, as when the clamp and cautery are employed. The formation of stricture is obviated.

The patient is given a cathartic and the tampon painlessly removed at the end of forty-eight hours. There is no pain nor bleeding with the movement of the bowels. After the bowels have moved the patient is instructed to keep them soft for two or three weeks by taking compound licorice powder or Apenta water, the latter being very palatable and effective. He has performed this operation in fifty cases, with more satisfactory results than he has obtained by any other method.—*Miss. Valley Med. Assn.*

